# Supplementary material for: Association between the Preoperative Dietary Antioxidant Index and Postoperative Quality of Life in Patients with Esophageal Squamous Cell Carcinoma: A Prospective Study Based on the TTD Model
Source: Nutrients. 2023 Jun 21;15(13):2828. doi: 10.3390/nu15132828 (PMC10343810; doi:10.3390/nu15132828)
Supplement: Supplementary file 1 [file nutrients-15-02828-s001.zip › nutrients-2425089-supplementary.pdf]

Supplement Table S1. The number of patients with deterioration in each domain at each follow-up time point and their percentage of all patients deteriorating in that domain.

domains	Follow-up times															
	1st	2nd	3rd	4th	5th	6th	7th	8th	9th	10th	11th	12th	13th	14th	15th	16th
QLQ-C30																
Global health status/QOL	16(5.16)	75(24.19)	53(17.10)	39 (12.58)	59(19.03)	23(7.42)	14(4.52)	10(3.22)	7(2.26)	9(2.90)	3(0.97)	1(0.32)	1(0.32)	0(0.00)	0(0.00)	0(0.00)
Physical functioning	11(3.38)	76(23.38)	46(14.15)	51(15.69)	70(21.54)	29(8.92)	13(4.00)	7(2.15)	6(1.85)	8(2.46)	2(0.62)	3(0.92)	0(0.00)	2(0.62)	1(0.31)	0(0.00)
Role functioning	11(3.72)	44(14.86)	38(12.83)	43(14.53)	69(23.31)	35(11.82)	17(5.74)	11(3.72)	8(2.70)	13(4.39)	1(0.34)	1(0.34)	3(1.01)	1(0.34)	1(0.34)	0(0.00)
Emotional functioning	5(19.2)	22(8.43)	33(12.64)	38(14.56)	73(27.97)	25(9.58)	20(7.66)	17(6.51)	9(3.45)	9(3.45)	4(1.53)	2(0.77)	2(0.77)	1(0.38)	1(0.38)	0(0.00)
Cognitive functioning	8(3.35)	22(9.21)	25(10.46)	27(11.30)	54(22.59)	35(14.64)	14(5.86)	10(4.18)	12(5.02)	18(7.53)	7(2.93)	3(1.26)	1(0.42)	1(0.42)	2(0.84)	0(0.00)
Social functioning	11(4.17)	37(14.02)	31(11.74)	39(14.77)	59(22.35)	25(9.470	19(7.20)	12(4.55)	8(3.03)	7(2.65)	3(1.14)	2(0.76)	6(2.27)	4(1.51)	1(0.38)	0(0.00)
Fatigue	12(4.15)	55(19.03)	50(17.30)	39(13.49)	65(22.49)	14(8.30)	15(5.19)	6(2.08)	9(3.11)	6(2.08)	1(0.35)	1(0.35)	2(0.69)	0(0.00)	3(1.04)	1(0.35)
Nausea/vomiting	10(3.68)	39(14.34)	44(16.18)	28(10.29)	48(17.64)	32(11.76)	20(7.35)	20(7.35)	12(4.41)	9(3.31)	2(0.74)	3(1.10)	2(0.74)	1(0.37)	2(0.74)	2(0.74)
Pain	12(4.67)	30(11.67)	37(14.40)	32(12.45)	53(20.62)	22(8.56)	19(7.39)	16(6.22)	13(5.06)	11(4.28)	5(1.95)	2(0.78)	3(1.17)	0(0.00)	2(0.78)	0(0.00)
Dyspnea	9(3.47)	31(11.97)	36(13.90)	35(13.51)	61(23.55)	26(10.04)	16(6.18)	12(4.63)	19(7.34)	7(2.70)	4(1.54)	2(0.77)	1(0.39)	0(0.00)	0(0.00)	0(0.00)
Insomnia	8(2.99)	31(11.57)	36(13.43)	28(10.45)	57(21.27)	31(11.57)	25(9.33)	14(5.22)	12(4.48)	10(3.73)	5(1.87)	7(2.61)	2(0.75)	0(0.00)	2(0.75)	0(0.00)
Appetite loss	9(3.36)	31(11.57)	32(11.94)	35(13.06)	63(23.51)	24(8.96)	22(8.21)	13(4.85)	15(5.60)	13(4.85)	6(2.24)	1(0.37)	2(0.75)	1(0.37)	1(0.37)	0(0.00)
Constipation	8(3.88)	14(6.80)	18(8.74)	18(8.74)	56(27.18)	26(12.62)	18(8.74)	16(7.77)	12(5.83)	9(4.37)	4(1.94)	0(0.00)	5(2.43)	1(0.49)	1(0.49)	0(0.00)
Diarrhea	10(3.95)	24(9.49)	30(11.86)	29(11.46)	63(24.90)	31(12.25)	23(9.09)	20(7.91)	10(3.95)	7(2.77)	3(1.19)	0(0.00)	2(0.79)	1(0.40)	0(0.00)	0(0.00)
QLQ-QES18																
Dysphagia	12(4.01)	63(21.07)	52(17.39)	48(16.05)	58(19.40)	38(12.71)	13(4.35)	5(1.67)	3(1.00)	1(0.33)	3(1.00)	0(0.00)	2(0.67)	1(0.33)	0(0.00)	0(0.00)
Eating problems	13(4.66)	48(17.20)	41(14.70)	30(10.75)	74(26.52)	23(8.24)	13(4.66)	10(3.58)	7(2.51)	16(5.73)	0(0.00)	0(0.00)	3(1.08)	0(0.00)	1(0.36)	0(0.00)
Reflux	13(4.09)	51(16.04)	37(11.64)	47(14.78)	71(22.33)	37(11.64)	18(5.66)	15(4.72)	12(3.77)	10(3.14)	3(0.94)	1(0.31)	0(0.00)	1(0.31)	1(0.31)	1(0.31)
Odynophagia	9(3.69)	25(10.25)	35(14.34)	31(12.70)	53(21.72)	27(11.07)	16(6.56)	14(5.74)	14(5.74)	10(4.10)	4(1.64)	1(0.41)	1(0.41)	1(0.41)	1(0.41)	2(0.82)
Trouble swallowing saliva	7(3.29)	15(7.04)	26(12.21)	22(10.33)	47(22.07)	32(15.02)	11(5.16)	20(9.40)	11(5.16)	11(5.16)	2(0.94)	3(1.41)	2(0.94)	2(0.94)	1(0.47)	1(0.47)

Cont.

domains	Follow-up times															
	1st	2nd	3rd	4th	5th	6th	7th	8th	9th	10th	11th	12th	13th	14th	15th	16th
QLQ-C30																
Choking when swallowing	9(3.80)	35(14.77)	36(15.19)	28(11.81)	44(18.57)	27(11.39)	11(4.64)	18(7.59)	9(3.79)	11(4.64)	3(1.27)	2(0.84)	1(0.43)	1(0.43)	1(0.43)	1(0.43)
Dry mouth	10(4.24)	22(9.32)	20(8.47)	29(12.29)	49(20.76)	26(11.02)	16(6.78)	15(6.36)	13(5.51)	14(5.93)	6(2.54)	5(2.12)	5(2.12)	1(0.42)	4(1.69)	1(0.42)
Trouble with taste	6(3.00)	20(10.00)	17(8.50)	19(9.50)	45(22.50)	29(14.50)	16(8.00)	14(7.00)	12(6.00)	10(5.00)	2(1.00)	3(1.50)	5(2.50)	1(0.50)	1(0.50)	0(0.00)
Coughing	15(6.49)	29(12.55)	28(12.12)	28(12.12)	47(20.35)	26(11.26)	15(6.49)	11(4.76)	16(6.93)	9(3.90)	3(1.30)	0(0.00)	3(1.30)	0(0.00)	1(0.43)	0(0.00)
Speech problems	6(2.80)	21(9.81)	22(10.28)	23(10.75)	49(22.90)	25(11.68)	17(7.94)	14(6.54)	15(7.01)	11(5.14)	1(0.47)	3(6.07)	5(2.34)	1(0.47)	1(0.47)	0(0.00)

Supplement Table S2. The incidence of TTD events in each dimension of the QLQ-C30/ QLQ-OES18 scale in ESCC patients with low and high DAI.

Domain/scale	DAI<0.729 [n (%)]	DAI≥0.729 [n (%)]	χ^2	*P* value
QLQ-C30				
Global health status/QOL	256 (83.4)	54 (78.3)	1.023	0.312
Functional scales				
Physical functioning	267 (87.0)	58 (84.1)	0.408	0.523
Role functioning	244 (79.5)	52 (76.5)	0.303	0.582
Emotional functioning	219 (71.3)	42 (60.9)	2.907	0.088
Cognitive functioning	201 (65.5)	38 (55.9)	2.215	0.137
Social functioning	217 (70.7)	47 (69.1)	0.066	0.798
Symptom scales				
Fatigue	239 (77.9)	50 (72.5)	0.919	0.338
Nausea/vomiting	223 (72.6)	49 (71.0)	0.074	0.785
Pain	211 (68.7)	46 (66.7)	0.111	0.739
Dyspnea	216 (70.4)	43 (63.2)	1.322	0.250
Insomnia	218 (71.0)	50 (72.5)	0.058	0.809
Appetite loss	219 (71.3)	49 (71.0)	0.003	0.958
Constipation	166 (54.2)	40 (58.0)	0.315	0.575
Diarrhea	206 (67.1)	47 (68.1)	0.026	0.871
QLQ-QES18				
General symptom scales				
Dysphagia	246 (80.7)	53 (76.8)	0.519	0.471
Eating problems	231 (75.7)	48 (70.6)	0.782	0.376
Reflux	259 (84.9)	59 (85.5)	0.015	0.901
Odynophagia	201 (65.9)	43 (63.2)	0.175	0.676
General symptom items				
Trouble swallowing saliva	175 (57.4)	38 (55.1)	0.122	0.727
Choking when swallowing	197 (64.6)	40 (58.0)	1.062	0.303
Dry mouth	199 (65.2)	37 (54.4)	2.808	0.094
Trouble with taste	165 (54.1)	35 (51.5)	0.154	0.694
Coughing	193 (63.3)	38 (55.1)	1.605	0.205
Speech problems	180 (59.0)	34 (49.3)	2.181	0.140
